# Development and validation of an Opioid Attractiveness Scale: a novel measure of the attractiveness of opioid products to potential abusers

**DOI:** 10.1186/1477-7517-3-5

**Published:** 2006-02-02

**Authors:** Stephen F Butler, Christine Benoit, Simon H Budman, Kathrine C Fernandez, Cynthia McCormick, Synne Wing Venuti, Nathaniel Katz

**Affiliations:** 1 Pain and Opioid Division, Inflexxion, Inc., 320 Needham St., Ste. 100, Newton, MA 02464, USA; 2 Department of Psychiatry, Harvard Medical School, 25 Shattuck St, Boston, MA 02115, USA; 3 McCormick Consultation, LLC, 9127 Friars Rd., Bethesda, MD 20817, USA; 4 Department of Anesthesiology, Tufts University School of Medicine, 136 Harrison Ave, Boston, MA 02111, USA

## Abstract

**Background:**

The growing trends in opioid abuse, assessment of the abuse liability of prescription opioid products, and growing efforts by the pharmaceutical industry to develop 'abuse-resistant' formulations highlight a need to understand the features that make one product more 'attractive' than another to potential abusers. We developed a scale to measure the 'attractiveness' of prescription opioids to potential abusers, and used the scale to measure the relative attractiveness of 14 opioid analgesic products.

**Methods:**

First, the concept of attractiveness was empirically defined with a group of prescription opioid abusers and experts in opioid abuse using a process called Concept Mapping. Abuse liability consisted of two components: factors intrinsic to the drug formulation (e.g., speed of onset, duration) and factors extrinsic to drug formulation (e.g., availability, availability of alternatives, cost). A 17-item Opioid Attractiveness Scale (OAS) was constructed, focusing on factors intrinsic to the drug product.

**Results:**

A total of 144 individuals participated in tests of validity and reliability. Internal consistency was excellent (Cronbach's α = 0.85–0.94). Drug rankings based on OAS scores achieved good inter-rater agreement (Kendall's W 0.37, p < 0.001). Agreement on drug OAS scores between the developmental sample and a confirmation sample was good (IntraClass Correlations [ICC] of 0.65–0.69). Global ratings of overall attractiveness of the 14 selected opioid products by substance abuse counselors corresponded with the rankings based on OAS ratings of the abuser group. Finally, substance abuse counselors completed the OAS, yielding a high level of correspondence with ratings by the abuser group (ICC = 0.83, p = 0.002). The OAS differentiated attractiveness among 14 selected pharmaceutical opioid products. OxyContin, Dilaudid, and Percocet were ranked highest (most attractive); Talwin NX and Duragesic were ranked lowest (least attractive).

**Conclusion:**

An initial examination of the psychometric properties of the OAS suggests that it is a valid and reliable scale. The OAS may be useful in providing important guidance on product features that are attractive to potential abusers.

## Background

The study of opiate abuse has a long history in the humanities and the social sciences, but a limited one in terms of clinical science and drug development studies [1-5]. Epidemiologic data (Substance Abuse and Mental Health Services Administration [SAMHSA], the National Institute on Drug Abuse's Monitoring the Future project, and the Drug Abuse Warning Network [DAWN]) indicate that non-medical use and abuse of prescription opioids is on the increase in the United States [6]. Left untreated, opiate dependence is responsible for significant morbidity and mortality. For example, use of illicit opiates is associated with an increased risk of hepatitis C infection, HIV infection, and other medical consequences such as overdose [7]. In addition, there has been an increase in unintentional deaths related to opioid medication in several areas across the United States [8,9].

Misuse and abuse of narcotics has been a subject of medical concern for many years [10-12]. To address this concern, a number of patents have been filed by pharmaceutical companies for 'abuse resistant' opioid formulations [13]. The pharmaceutical industry continues to develop drugs with enhanced delivery capabilities [14-17]. Some of these new formulations have been assumed to be relatively abuse-resistant, yet their real potential for abuse is unknown. Given the continuing developments in new products and formulations reaching the market, health professionals and those involved in the care and treatment of substance abusers need a way of assessing the relative attractiveness of these new products in order to understand and manage any associated risks to society. There has been no work done to date examining the qualities that make a specific prescription opioid product more attractive, or more unattractive, to potential recreational users/abusers.

To address this need, an Opioid Attractiveness Scale (OAS) was developed and validated. The OAS is offered as a sensitive and reliable method for detecting differences in attractiveness of different prescription opioid products and formulations to potential abusers.

## Methods

The development process consisted of four phases. The first was to define what makes different prescription opioid products attractive or unattractive to potential abusers, and hence to establish the content of the scale. The second phase was to generate and evaluate items for the scale using Concept Mapping. Items generated in this way underwent a conceptual evaluation process and pilot testing. An alpha version was constructed and examined for usability in a small pilot. During phase three, a beta version was then empirically evaluated for reliability and validity on a developmental sample of substance abuse clients. In phase four, a final version of the OAS was subjected to cross validation with a new sample of substance abuse clients and validation against ratings made by professionals who treat prescription opioid abuse.

### Participants

Participants (stakeholders) included casual users, substance abuse clients, pain patients, impaired professionals, and opioid and substance abuse experts (Table 1). Experts were recruited through professional referrals. The remaining stakeholders were recruited via provision of flyers in pain, methadone, and substance abuse clinics throughout the United States, and via internet advertisements. Interested potential participants called a toll-free number to obtain additional information about the study. Using a standardized screener, a research team member screened all callers. Individuals who consented and met specific inclusion and exclusion criteria were involved in the study.

**Table 1:** Study phases and participants

**Study phase**	**Participant category**
Phase1: Pre-Concept Mapping	Pre-Concept Mapping sample: • Casual users • Substance abuse clients • Pain patients • Impaired professionals • Substance abuse experts
Phase 2: Concept Mapping, usability testing	Concept Mapping sample: • Casual users • Substance abuse clients • Pain patients • Impaired professionals • Substance abuse experts
	Usability testing sample: • Substance abuse clients • Pain patients
Phase 3: Initial evaluation	Developmental sample: • Casual users • Substance abuse clients
Phase 4: Assessment of reliability and validation of scale	Confirmation sample: • Casual users • Substance abuse clients
	Validation sample: • Substance abuse experts
Definitions of participant categories Casual users: active recreational users of prescription opioids who were not in treatment Substance abuse clients: persons in treatment for opioid abuse Pain patients: patients with a history of opioid misuse Impaired professionals: persons in the medical field who were current or previous prescription opioid abusers Substance abuse experts: professional substance abuse counselors experienced in treating opioid addiction	

### Phase 1: Pre-Concept Mapping

The first step in Concept Mapping involves open-ended interviews with various stakeholders who were scheduled for a one-hour interview with research team members. Interviews were conducted by telephone using a structured script designed to capture all of the factors that make different formulations of prescription opioids attractive and unattractive to individuals with a propensity for substance abuse. Notes and tape recordings of interviews were reviewed by the team. The qualitative results of these interviews were used to inform the focus prompts in the next stage of Concept Mapping.

### Phase 2: Concept Mapping phase and creation of scale

Concept Mapping is an inductive but structured process in which participants, through brainstorming, generated a list of specific statements in response to prompts/questions about the attractiveness of prescription opioid products (developed from the pre-Concept Mapping phase). Participants were then re-contacted to sort the statements into conceptual groups and rate each statement using a Likert-like scale. In this case, statements were rated as to their importance to attractiveness. Participants completed this activity by mail or e-mail. Materials were accompanied by detailed instructions and a follow-up phone call by a research team member. Using multidimensional scaling and cluster analysis, Concept Mapping moves from the list of statements, in a stepwise fashion, toward more general concepts. This technique allows the transformation of qualitative data into a form suitable for rigorous statistical examination. A pilot scale (alpha version) was developed as a result of this process.

The alpha version of the OAS was pilot tested with a small group of stakeholders. The purpose of this exercise was to test the language of the OAS to make certain respondents could comprehend the content and understand directions of the activity. A research team member conducted the structured pilot interview with each participant. Participants were asked to use the alpha OAS to judge the attractiveness of five opioids. Reference cards containing a picture, the brand name, street names, and other product-specific information was provided for each opioid as visual cues. This pilot test resulted in modifications to the OAS yielding a beta version.

### Phase 3: Initial evaluation of the beta OAS

**Figure 1:**
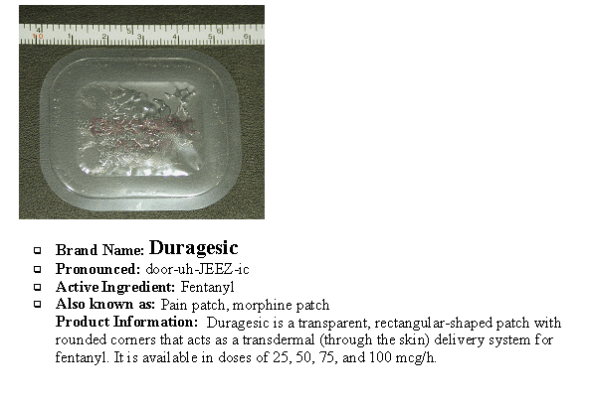
A sample opioid information card.

The beta OAS was evaluated empirically with a group of potential abusers in order to assess the replicability and reliability of the scale. Ratings were conducted in face-to-face meetings or remotely (using mailed versions of the materials accompanied by detailed instructions and a follow-up phone call from a research team member). Participants were provided with the OAS rating materials including an information card for 14 opioid products and a separate 17-item rating scale for each drug. They then rated how attractive thespecified feature of the drug product was to them using a five-point scale (1 – 'this feature makes the drug extremely unattractive to try'; 2 – 'this feature makes the drug somewhat unattractive to try'; 3 – 'this feature does not affect my interest in trying the drug'; 4 – 'this feature makes the drug somewhat attractive to try'; 5 – 'this feature makes the drug extremely attractive to try'). The word 'try' was featured in the scale instead of the word 'use', in order to determine what would attract new users, including casual users, to sample new products. Furthermore, this wording allowed for the application of the OAS to products that, at the time of this study, were not available in the market, as demonstrated with the fentanyl matrix patch. Participants used the scale to judge the attractiveness for abuse of 14 prescription opioid products (Actiq, Avinza, Dilaudid, Duragesic, Fentanyl matrix patch, Kadian, MS Contin, OxyContin, Percocet, Stadol Nasal Spray, Suboxone and Talwin NX) shown to them in pictorial form on information cards (Figure 1), including a transdermal formulation of fentanyl in a drug-in-adhesive, matrix patch formulation, not then marketed in the United States. These 14 products were chosen to accommodate the following goals: to include the most commonly prescribed opioids in the United States; to include drug products felt to represent a spectrum of abuse liabilities; to include both immediate- and sustained-release products; and to include drugs designed and perceived to be relatively abuse-resistant (e.g. Talwin-NX and Suboxone). Oxycodone combination products were felt to be similar enough to hydrocodone combinations with respect to abuse liability so that only the latter were included.

The information cards were developed by a team of three pharmacists and two physicians, who utilized information in the public domain to specify the features of each drug required for each item of the OAS (e.g., onset of action, duration of effect, etc).

### Phase 4: Cross validation and criterion validation of the scale

An important aspect of scale development is cross validation, a necessary step to ensure that the results of the ratings and rankings achieved during scale construction are replicable and not simply an artifact associated with a particular sample. Thus, the procedure was repeated with a new group of potential abusers. The assessment methods for this group of substance abuse clients were identical to those used with the developmental sample. In addition, it was considered important to establish some 'outside' criterion against which to compare the substance abusers' OAS ratings. Since there is no existing 'gold standard', we compared results obtained by clients with ratings of the same drugs made by professional substance abuse counselors experienced in treating opioid addiction.

### Statistical methods

Internal consistency of the OAS was assessed using standard procedures for calculating coefficient α [18]. Agreement on the extent to which the various drugs are 'attractive' was calculated two ways. First, agreement on the relative ranking of the medications was calculated by ordering drugs according to their OAS score and using Kendall's Coefficient of Concordance statistic (Kendall's W) [19] to determine whether the concordance was significant. Secondly, comparison of the extent to which different groups assigned similar OAS scores to the same drug were calculated using the IntraClass Correlation (ICC) statistic, an accepted method for examining the correspondence of the ratings of two or more raters judging the same items. Confirmation of the OAS was established by calculating the ICC across all drugs being evaluated in order to demonstrate that the output of the scale is replicable. As an additional test of validity, agreement between rankings by potential abusers and those by substance abuse counselors was assessed using the Kendall's W test. The study was approved by the Inflexxion Institutional Review Board.

## Results

### Participants

A total of 144 individuals (opioid users and professionals) participated in the development and testing of the OAS. Study participants were recruited predominantly from the United States and included casual prescription opioid users (active recreational users who were not in treatment); substance abuse clients (persons in treatment for opioid abuse); pain patients with a history of opioid misuse; impaired professionals (persons in the medical field who were current or previous prescription opioid abusers); and experts (professional substance abuse counselors experienced in treating opioid addiction; Table 2).

**Table 2:** Study participants and demographics

	**Number (%) of participants**				
	
**Characteristic**	**Pre-Concept Mapping**	**Concept Mapping**	**Developmental sample**	**Confirmation sample**	**Substance abuse experts (validation) sample**
Total number of participants	16	36	38	42	12
Age (years) Mean ± SD	39.08 ± 9.38	38.2 ± 9.0	39.3 ± 12.2	37.8 ± 9.7	45.5 ± 8.1
Gender	8 (50) male	10 (33) male	22 (58) male	27 (64) male	6 (50) male
	8 (50) female	20 (67) female	15 (39) female	13 (31) female	6 (50) female
			1 (3) missing	2 (5) missing	
Race					
- White	15 (94)	22 (73)	23 (61)	23 (55)	9 (75)
- African-American	1 (6)	2 (7)	3 (8)	6 (14)	0
- Hispanic	0	5 (17)	10 (26)	11 (26)	3 (25)
- Other	0	1 (3)	2 (5)	1 (2)	0
- Missing	0	0	0	1 (2)	0
Residence					
- Urban	12 (81)	25 (83)	28 (74)	33 (79)	5 (42)
- Rural	3 (19)	5 (17)	10 (26)	8 (19)	7 (58)
- Missing	0	0	0	0	0
Casual users	3 (19)	7 (19)	8 (21)	9 (21)	0
Substance abuse clients	2 (12)	14 (39)	30 (79)	33 (79)	0
Pain patients	4 (25)	6 (17)	0	0	0
Impaired professionals	3 (19)	3 (8)	0	0	0
Substance abuse experts	4 (25)	6 (17)	0	0	0

Sixteen key stakeholders were recruited for Phase 1, including four opioid abuse experts, three impaired professionals, three casual prescription opioid users, two persons in treatment for opiate abuse, and four pain patients with a history of opioid misuse. Thirty-six stakeholders were recruited and completed Concept Mapping phases, including six pain patients, seven casual users, 14 substance abuse clients in treatment, three impaired professionals, and six substance abuse/opioid experts. Four individuals participated in the usability testing of the alpha version of the OAS. Face-to-face usability of the scale was tested using two substance abuse clients and two pain patients from various socio-economic and racial/ethnic backgrounds.

### Pre-Concept Mapping

Eight key areas were consistently reported to be an indicator of attractiveness. The most prevalent theme was the *quality of the high*, with participants reporting that the type of high they were seeking would inform their choice of prescription opioid. *Duration of the high *was also an attractive quality, as was the preference for prescription opioids in pill form which was considered to be more convenient than other delivery systems due to ease of swallowing, chewing, or crushing and snorting. A further factor influencing attractiveness was *availability *(via prescription, street or 'club' supply), the choice of prescription opioid dependent on what medications would be available at any point in time.

*Cost *was another component of the attractiveness of a product for abuse for many subjects; although some participants claimed that cost was not an issue, this appeared to be because they received free medications or because money in general was not an issue for them. *Side effects *and *withdrawal effects *were also considered important factors, as was *peer influence*, which seems to inform users which products are attractive and which should be avoided. Finally, the *real or perceived dangers *associated with a drug were a factor influencing attractiveness, but were not considered a deterrent by some of the long-term prescription opioid abuser participants.

### Concept Mapping and development of the scale

In the brainstorming phase, the participants generated over 2000 separate statements regarding factors that make opioids attractive or unattractive. These statements were reviewed to remove duplicates and nonsensical entries and to combine similar statements. As a result of this process, 109 statements were retained for use in the sorting and rating phase (see example statements in Table 3).

**Table 3:** Examples of statements generated by the Concept Mapping process

This form of opioid medication makes you nauseous This form of opioid medication results in a high that allows you to function (go to work, drive etc) This form of opioid medication doesn't affect your appearance This form of opioid medication can be safely injected This form of opioid medication is preferred by my friends This form of opioid medication makes you indifferent This form of opioid medication has a greater addiction potential This form of opioid medication gives you a whole-body buzz/rush This form of opioid medication is painful to use This form of opioid medication makes me feel like I can do anything This form of opioid medication makes me feel I can do more than usual This form of opioid medication makes you excited and hyper This form of opioid medication makes you feel sleepy This form of opioid medication makes you incoherent (cannot communicate with others) This form of opioid medication can be easily concealed or hidden This form of opioid medication creates pleasant hallucinations This form of opioid medication creates tolerance easily (must take more to get high) This form of opioid medication heightens sexual feelings This form of opioid medication makes you thirsty ('cotton mouth') This form of opioid medication relieves your anxiety

The sorting and rating process resulted in the definition of three dimensions of the scale: positive features of the preparation, negative features of the preparation and social milieu effects of the preparation. To clarify, in this project, social milieu was operationally defined to include such phenomena as the product's availability, cost, and possible stigmas that vary among geographic locations and social networks.

Based on average ratings of importance for the statements within each dimension, the most important of the three dimensions (mean rating for dimension = 3.83) was the positive features of the preparation (e.g., statements such as 'can be safely injected', 'easily dissolved', 'long-lasting high' and 'multiple uses can be obtained from one unit'). A mean rating of 3.57 was obtained for social milieu effects of the preparation (e.g., statements such as 'easily concealed or hidden', 'easy to get from pharmacy, doctor, street', 'a lot of information is available on the internet', and 'less likely to lead to arrest or bust'). The dimension characterizing negative features of the formulation was rated the lowest with a mean rating of 2.94.

### Alpha version creation and pilot usability testing

Based on the Concept Mapping results, an alpha version of the OAS was created with 22 items reflecting the three dimensions of positive and negative features of the product preparation, as well as the social milieu dimension. The usability testing resulted in some modifications including: providing layman's definitions of certain words, clarifying the DEA classifications of drugs; reducing the reading grade-level to 7.5 (Flesch Kincaid Grade Level); and, removing 'social milieu' items due to lack of agreement among the raters and the potential influence of changing context. While social milieu-type questions may carry important variance with respect to how individual raters view the attractiveness of various medication preparations, such items may also reflect situational variables not directly tied to qualities of the product itself. Thus, whether a product is easy or difficult to obtain at a pharmacy may be an important consideration on the street, but it is not necessarily related to the product itself, and may change from time to time or place to place. Additionally, social milieu items are very subjective, and can vary between stakeholder populations and within stakeholder groups. Finally it was determined, based on participant feedback, that participants should be presented with pictures of the opioid to assist them in the rating activity by providing a visual cue (see Figure 1). Brand and street names were also included on the opioid information card based on the results of usability testing. The modified beta version of the OAS was thus reduced to 17 items (see Table 4).

**Table 4:** Content of OAS items: each is rated on a five-point scale

1	Painful to snort or inject
2	Ability to conceal or hide
3	Duration of withdrawal symptoms
4	Messy to use
5	Ability to change into another form for recreational use
6	Presence of toxic metabolites
7	Solubility in water, vinegar, alcohol, etc
8	This medication's duration of effect
9	Short onset (works quickly)
10	Potency compared to morphine
11	Contains waxes, gums, binders, fillers or other impurities
12	Contains an opioid antagonist
13	Divisible into smaller doses
14	Ability to use in different ways (snort, smoke, eat, IV, etc) to get different highs
15	Designed in a way that is difficult to abuse to get a high
16	This medication comes in high doses
17	Drug considered to have high (or low) potential for abuse (DEA classification)

Note: The specific item for each drug is worded so that a higher rating reflects a greater degree of 'attractiveness.'

Stakeholders used the beta version of the OAS to rate 14 opioid products. Although all participants reported using at least one of the 13 actual drugs (see Table 5, excluding the hypothetical transdermal matrix patch formulation of fentanyl), raters did not need to have experience with every drug. Rather, ratings were based on information provided to the participants on the information cards. Raters took an average of 5 minutes to complete each scale.

**Table 5:** Mean ranks of drugs: OAS ratings in descending order for development sample using two scoring approaches

**Product order for mean composite**	**Mean rank for mean composite**	**Product order for sum composite**	**Mean rank for sum composite**
Vicodin	11.94	Vicodin	11.94
OxyContin	10.39	OxyContin	10.39
Talwin NX	10.29	Talwin NX	10.27
MS Contin	9.39	MS Contin	9.41
Methadone	9.08	Methadone	8.89
Dilaudid	7.42	Dilaudid	7.45
Actiq	7.12	Actiq	7.14
Avinza	6.83	Avinza	6.86
Kadian	6.73	Kadian	6.74
Percocet	6.47	Percocet	6.52
Suboxone	6.32	Suboxone	6.32
Fentanyl matrix patch	5.52	Fentanyl matrix patch	5.50
Stadol Nasal Spray	4.52	Stadol Nasal Spray	4.58
Duragesic fentanyl reservoir patch	2.98	Duragesic fentanyl reservoir patch	2.98

### Scoring of the scale (development sample)

A number of different methods of obtaining an overall OAS score were considered, including the sum of the items scored, the mean score, Z-score transformation, calculation based on attractive items only and a weighted composite. In order to select from these options, two criteria were used: agreement among the raters and the extent to which the scoring methods differentiated the drugs. In general, the various composite scores yielded similar information (intercorrelations of the different scores were all >0.80, and all but one were ≥0.88). The sum and mean composite scores, however, best met the criteria.

Calculation of mean composite scores from 38 raters showed that means ranged from a high of 3.73 for Vicodin (most attractive) to a low of 2.58 for Duragesic (least attractive). Table 5 shows the mean ranks of the various medications for all 38 raters. To arrive at these ranks, a ranking of all 14 drugs from least to most attractive (1 to 14) was developed for each individual rater based on his or her composite scores for the drug. The mean rank was then calculated and ordered. Again, this process resulted in Vicodin receiving the highest rank (11.94) and Duragesic the lowest (2.98). Rankings based on the sum scores of the various medications are similar.

### Cross validation and criterion validation of the scale (confirmation sample)

Reliability (internal consistency) was calculated on data collected from a new, confirmation sample of 42 substance abusers (see Table 2). The appropriate level of internal consistency is determined by convention, and a coefficient α of ≥0.70 is generally considered good [18]. In this study, internal consistency of the OAS was calculated for each drug and was found to be excellent (α = 0.85–0.94).

Cross-validation of the reliability of the scale as well as the ratings and rankings produced by the OAS is an important part of the validation process. Ratings of the confirmation sample resulted in the rankings shown in Table 6. It is evident that the order of the drugs was different from the initial rankings described above. OxyContin had the highest attractiveness score for both mean and sum composite values, but Duragesic remained the least attractive of the products rated.

**Table 6:** Mean ranks of drugs based on OAS ratings in descending order for confirmation/cross-validation sample

**Product order for mean composite**	**Mean rank for mean composite**	**Product order for sum composite**	**Mean rank for sum composite**
OxyContin	12.05	OxyContin	12.05
Avinza	10.03	Avinza	10.06
Actiq	8.88	Actiq	8.69
Methadone	8.58	Methadone	8.64
Dilaudid	8.45	MS Contin	8.42
MS Contin	8.31	Dilaudid	8.27
Kadian	7.55	Percocet	7.53
Percocet	7.44	Kadian	7.50
Vicodin	7.38	Vicodin	7.39
Fentanyl matrix patch	6.55	Fentanyl matrix patch	6.34
Talwin NX	6.13	Talwin NX	6.25
Stadol Nasal Spray	5.72	Stadol Nasal Spray	5.75
Suboxone	5.45	Suboxone	5.53
Duragesic fentanyl reservoir patch	2.50	Duragesic fentanyl reservoir patch	2.58

Despite changes in the ranks of individual drugs between the developmental and confirmation samples, the level of agreement on the actual scores assigned to the individual drugs was examined using the ICC statistic. Traditionally, interpretations of the magnitude of ICCs assume that values >0.80 represent 'perfect' agreement; 0.61–0.80 is substantial; 0.41–0.60 is moderate; and 0.21–0.40 is fair reliability [20]. For the mean composite score the ICC was 0.65 (p = 0.037); for the sum composite score, the value was 0.69 (p = 0.022). These results are both significant and reflect substantial agreement, suggesting that the results of the OAS were replicated in the cross validation.

In order to relate OAS scores assigned by a group of potential abusers to some outside criterion, rankings made by substance abusers were compared with rankings of the drugs made by substance abuse counselors. Reasonably good correspondence was found between the two groups. Kendall's W was 0.89 (p = 0.039) for the mean composite scores and 0.87 (p = 0.048) for the sum composite scores. This suggests that the relative rankings of the drugs by the two groups were comparable. Similarly, direct comparisons of counselors' and substance abusers' ratings showed very high agreement (ICC = 0.83, p = 0.002). Considering that these raters were not trained to agree, this level of agreement is very high. Taken together, these data suggest that the OAS provides a reasonably robust measure of the relative attractiveness for abuse of specific opioid analgesics.

### Further examination of the drug ratings

**Figure 2:**
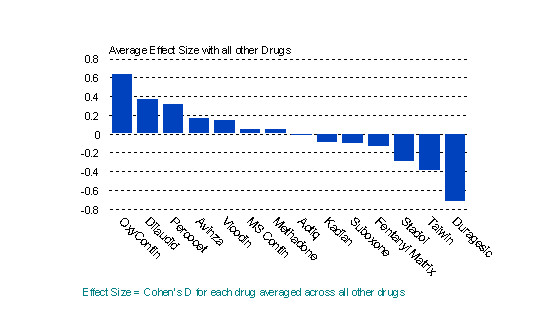
Average effect size for each drug with all other drugs.

In order to examine the clinical meaningfulness of differences in attractiveness ratings, the average effect size (Cohen's D) of each drug, as rated by substance abusers, was compared to every other drug rated.Drugs with small average effect sizes (approximately 0.20) are more similar to other drugs in their ratings, while those with large average effect sizes (approximately 0.80) are more likely to be different from most other drugs. Figure 2 shows a striking difference at the extremes, particularly between OxyContin and Duragesic. Clearly, OxyContin was viewed as much more attractive than the other drugs, while Duragesic is much less attractive to this group of abusers than all of the other medications investigated.

**Figure 3:**
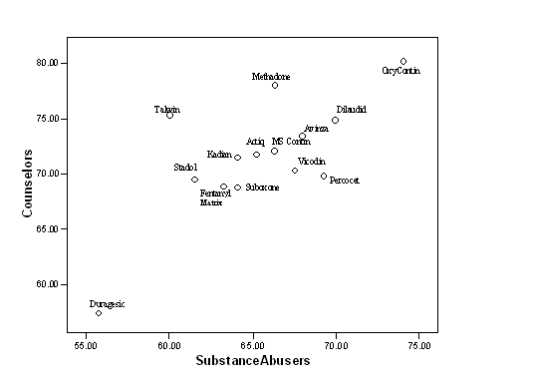
OAS ratings for substance abusers plotted against counselor ratings.

Comparison of the ratings of potential substance abusers with those of the counselors reinforced the extreme positions of OxyContin and Duragesic as the most and least attractive for abuse, respectively (Figure 3).

## Discussion

The OAS was developed empirically from patient and professional input. The scale development process included content validity, initial empirical testing, cross validation, and comparison with an outside criterion. Results indicate that the OAS appears to be a reliable and valid measure of the attractiveness for abuse of opioid analgesic products, yielding stable ratings and rankings of 14 prescription opioid medications that resonate with epidemiologic data and clinical experience [21].

The OAS ratings of the 14 opioid products (13 marketed products and one non-marketed product in the United States) showed marked differences in their relative attractiveness for abuse. OxyContin was considered the most attractive of the products rated, while Duragesic was considered the least attractive of the products rated. Interestingly, the fentanyl matrix patch rated as part of the validation process was considered relatively more attractive for abuse than the reservoir formulation used in Duragesic, even though the active ingredient (fentanyl) and route of delivery (transdermal) were the same.

The OAS demonstrated excellent reliability (internal consistency) and validity in the study samples. It is important to consider that, in this study, agreement among raters (typically considered reliability) was itself taken as evidence of validity. Agreement among observers is an accepted procedure to establish validity in psychometric questionnaire development where no objective 'gold standard' or external reference exists against which to measure a novel measurement tool [19]. Validity was further supported by high levels of agreement between heterogeneous groups of stakeholders (substance abusers and professional counselors), suggesting that the OAS provides a robust metric of what makes a prescription opioid attractive for abuse, and permits comparisons of attractiveness ratings for different products. That the counselors' rankings agreed highly with those of the substance abusers provides additional confirmation of the external validity of the ratings. We considered comparing OAS ratings to reported abuse rates as a "gold standard" criterion measure, but decided against this approach since there is no accepted method for ranking the abuse rates of marketed opioids based on available data. Also, our findings suggested that actual abuse rates will relate to factors intrinsic to the product, measured by the OAS, as well as factors extrinsic to the product, not captured in the OAS. Future research could productively explore predicting actual abuse rates by combining the OAS with a measure of these extrinsic factors.

The Concept Mapping process identified three dimensions relevant to the abuse liability of an opioid. Two dimensions highlighted aspects intrinsic to the drug product: positive features of the drug preparation (e.g., speed of onset, duration of effect, extractability) and negative features of the preparation (e.g. presence of impurities, presence of antagonist, messy). The third dimension, called social milieu features of the drug, tapped factors extrinsic to the drug product (e.g., availability, availability of alternatives, cost, social stigma). Pilot usability testing suggested substantial inter-rater variability of items reflecting these social milieu factors. That is, the social milieu items seemed sensitive to variation across individuals, time, and geography. Considerable work has been devoted to the symbolic meaning attributed to specific drugs by those who misuse them [22]. An understanding of such symbolic meaning will ultimately be necessary to comprehend psychological dependence on drugs. However, the team determined that the OAS would reflect only factors identified as intrinsic to the drug preparation. This decision was based on a consideration of several issues. One issue was that a goal of the OAS was to evaluate specific features of new products that are either newly on the market or even not yet available. Comparisons of such drugs to established, well-known drugs may be problematic. Another issue, at this early stage of scale development, was the goal of maximizing inter-rater agreement. It was felt that the introduction of items known to vary widely among individuals would adversely affect the development and psychometric properties of the OAS. This decision, however, does not imply that we consider social milieu questions to be irrelevant. Rather, it may make more sense to explore this important and complex issue with a separate scale. Nor does this decision suggest that we believe the OAS to be immune to the effects of social milieu on its ratings. Clearly, it is possible that omission of items tapping social milieu may introduce a threat to construct validity called 'construct under-representation' [23]. However, our data suggest that inclusion of this dimension may seriously hamper the OAS's reliability and ranking agreement among the various stakeholder groups which may itself introduce other, potential threats to validity. Future studies will be necessary to define more completely and to tease out the potentially differential effects of various aspects of social meaning on drug selection by those who misuse these substances.

Additionally, the reliability of substance abusers is in question. The data contain some suggestions that our sample of substance abusers may not have been entirely truthful or perhaps not taken the rating task as seriously as they might have. For example, nearly a quarter of both the developmental and the confirmation samples claimed experience with the fentanyl matrix patch, which had not been available at the time of the study. Whether this is due to a lack of seriousness about the task, misunderstandings of the research materials, a tendency of substance abusers towards braggadocio, or some other explanation is unknown, but such findings raise caution with respect to other indications of fact or opinion. Of course, these individuals and their ratings of the drugs are reasons for the OAS. To remedy this, it is suggested to include as many substance abusers as possible in future OAS research.

Finally, a note is in order regarding our claim to validity of the OAS. Quantifying the construct, "attractiveness for abuse" of medication preparations, has not been attempted before. This article presents an initial effort to provide such a measure. Careful attention was paid to content validity, using Concept Mapping procedures, inter-rater reliability, internal consistency, criterion validity and cross validation. Based on these analyses, we feel confident in claiming that the OAS has good reliability and validity. Nevertheless, the relative ranking of individual pharmaceutical products produced by the OAS may vary according to as yet uninvestigated threats to external validity (e.g., construct-irrelevant variance), such as raters' direct experience with a drug or an increased media profile for a product. Future research could usefully investigate the nature and impact of such variables on OAS scores.

While the OAS has some limitations, for now, however, it is a significant step forward in improving our understanding of the attractiveness of opioid medications for abuse. Providing industries and regulatory agencies with a scale that can be applied systematically to products in development can help address opioid risk management issues faced by these industries. Furthermore, health professionals can apply the OAS to products already in the market in order to understand better the relative attractiveness of new products to substance abusers and hence care for their clients. The OAS may potentially play an important role in reducing the harmful use of future prescription opioid products by individuals.

## Conclusion

The OAS represents a significant development in assessing what makes a prescription opioid product attractive or unattractive for abuse, and for identifying differences in abuse potential between different products. Such knowledge may help guide the rational development of abuse-resistant medications, or provide support for decision makers on how best to regulate prescription opioid products.

Use of the OAS to assess the attractiveness of established and new prescription opioid products, including modified-release forms, may provide information of value in informing the assessment of the relative risks of these products for abuse. We anticipate that the information generated will be an important first step in developing initiatives to manage this risk.

## Competing interests

This study was supported by Janssen Pharmaceutica, Inc.

## Authors' contributions

SFB contributed to all aspects of conceptualization, design, conduct, analyses and write-up. NK contributed to all aspects of conceptualization, medical content, design, analyses and interpretation. KCF contributed to conceptualization, design, implementation and write-up. CB contributed to implementation, recruitment, data collection and management. SHB contributed to conceptualization, design, conduct, analysis, interpretation and write-up. SWV contributed to conceptualization, design, implementation and write-up.
